# Divergent strigolactones or karrikins signaling is mediated by preferred AtD14/KAI2-MAX2-SMXL complex targeting specific SMXL domains

**DOI:** 10.1016/j.isci.2026.116062

**Published:** 2026-06-01

**Authors:** Miao Xie, Xinbei Xu, Meng Zhang, Huihuang Chen, Li Chen, Ruifeng Yao

**Affiliations:** 1 State Key Laboratory of Chemo and Biosensing, Hunan Provincial Key Laboratory of Plant Functional Genomics and Developmental Regulation, College of Biology, Longping Agricultural College, Hunan University, Changsha 410082, China; 2 Yuelushan Laboratory, Changsha 410082, China; 3 Hunan Research Center of the Basic Discipline for Cell Signaling, College of Biology, Hunan University, Changsha 410082, China

**Keywords:** Plant biology, Strigolactone signaling, Karrikin signaling, Receptor, SMAX1, SMXL7, Degradation

## Abstract

Strigolactones (SLs) and karrikins (KARs) are structurally related signaling molecules that regulate different aspects of plant development. In *Arabidopsis*, both SL and KAR signaling require the F box protein MAX2 but are perceived by different receptors, AtD14 and KAI2, respectively. These signals target distinct SMAX1-LIKE (SMXL) repressors, the SMXL7 and SMAX1 clade, respectively, for degradation. However, the mechanism underlying selective SMXL degradation remains elusive. Here, we propose that selective SMXL degradation arises from RGKT motif-dependent ternary protein complexes involving MAX2 and the respective receptors. Domain-swapping analysis between SMAX1 and SMXL7 indicates that the D1M (D1+M) and D2 domains contribute to hormone responsiveness in *Arabidopsis* through targeted degradation. Biochemical and structural analyses highlight the D2 domain and its RGKT motif as central to ternary complex formation. Our findings provide insights into how a single E3 ubiquitin ligase recruits distinct substrate proteins via different signaling receptors to achieve specific signal transduction.

## Highlights

• MAX2 recruits SMXL7 via SL-activated AtD14, and SMAX1 via KAR-activated KAI2
• SMXL7 and SMAX1 enter hormone-specific complexes via D2 domains and the RGKT motif
• AtD14/KAI2 interacts with specific SMXL D1M domain to facilitate SMXL degradation

## Graphical abstract

**Figure:**
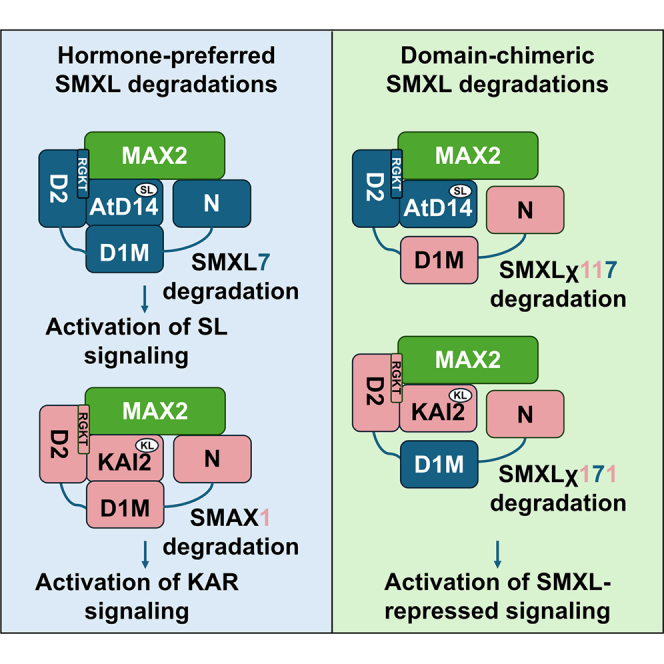

## Introduction

Strigolactones (SLs) and smoke-derived karrikins (KARs) are structurally related butenolides that regulate distinct aspects of plant growth and development.^1^^,^^2^ Initially identified as rhizosphere signaling molecules exuded by roots,^3^ SLs stimulate germination of parasitic plants and promote hyphal elongation of arbuscular mycorrhizal fungi, thereby facilitating symbiosis.^4^ They also function as plant hormones to modulate shoot branching, plant height, root architecture, leaf shape, flowering time, and stress responses.^5^^,^^6^^,^^7^^,^^8^^,^^9^ KARs, discovered in smoke from combusted plant matter,^10^ are critical for promoting primary seed germination, regulating seedling development and photosynthesis, and establishing arbuscular mycorrhizal symbiosis.^11^^,^^12^^,^^13^

In *Arabidopsis thaliana*, the perception mechanisms of SLs and KARs are highly conserved. The F box protein MORE AXILLARY GROWTH2 (MAX2), a component of the Skp-Cullin-F-box (SCF) type E3 ubiquitin ligase complex, is essential for both SL and KAR responses.^14^^,^^15^^,^^16^ The α/β hydrolase KARRIKIN INSENSITIVE2 (KAI2) is required for KAR perception and sensing of the yet-unidentified endogenous KAI2 ligands (KL),^17^^,^^18^ while its paralogs DWARF14 (OsD14) in rice (*Oryza sativa*) and AtD14 in *Arabidopsis* act as SL receptors.^17^^,^^19^^,^^20^ KAI2 paralogs in parasitic weeds were also identified as SL receptors that regulate seed germination, such as HYPOSENSETIVE TO LIGHT in *Striga hermonthica* (ShHTLs). Notably, these SL receptors mediate SL-induced germination that differs from KAI2 and AtD14.^21^^,^^22^ AtD14 (and its orthologs) and ShHTL7 (a representative SL receptor in weeds with high sensitivity) directly bind ligands, catalyze hydrolysis to generate a covalent modification at the catalytic His residue, and subsequently undergo conformational changes that enable interaction with MAX2.^19^^,^^23^^,^^24^^,^^25^ By contrast, the *in vitro* activation of KAI2 by KARs has shown variable results, while the stereoisomer GR24^*ent*−5DS^ (a part in the commonly used SL analogue *rac*-GR24) and some demethylated derivatives, such as dGR24^*ent*−5DS^ and dGerminnone, exhibit higher *in vitro* activity.^11^ SUPPRESSOR OF MAX2 1 (SMAX1) and SMAX1-LIKE2 (SMXL2) repress KAR signaling,^26^^,^^27^ whereas DWARF53 (D53) in rice and SMXL6, SMXL7, and SMXL8 in *Arabidopsis* repress SL signaling.^28^^,^^29^^,^^30^^,^^31^ SLs promote ubiquitination and proteasome-dependent degradation of SMXL6/7/8 in an AtD14- and MAX2-dependent manner, while KARs (and KL) trigger degradation of SMAX1 and SMXL2 via MAX2 and KAI2.^32^^,^^33^^,^^34^

SMXL proteins act as signaling hubs that regulate plant development by repressing target gene expression through modulating transcription factor activity, direct DNA binding, or chromatin remodeling.^35^^,^^36^^,^^37^^,^^38^^,^^39^ Their stability is regulated not only by SLs or KARs/KL but also by environmental cues (e.g., light, temperature, nutrient availability).^40^^,^^41^^,^^42^ In flowering plants, SMXLs are classified into four subgroups: aSMAX1, SMXL3/9, aSMXL4, and SMXL7/8; SMXL3/9 and SMXL7/8 are absent in gymnosperms.^43^ In *Arabidopsis*, SMAX1 and SMXL2 belong to the aSMAX1 clade (responsive to KARs/KL), while SMXL6/7/8 cluster in the SMXL7/8 clade (responsive to SLs). By contrast, SMXL3 (SMXL3/9 clade) and SMXL4/5 (aSMXL4 clade) are insensitive to SLs and KARs/KL, presumably due to the lack of an RGKT motif.^44^^,^^45^

A recent study showed that functional diversity between SMAX1 and SMXL7 arises from differences in protein activity rather than expression patterns.^46^ SMXL proteins contain four major domains: an N-terminal domain with a double Clp-N motif, and two putative ATPase domains (D1 and D2) linked by a central M domain.^29^^,^^47^ The N domain is the “output” domain determining developmental control by SMAX1 and SMXL7.^46^ The D2 domain is necessary and sufficient for hormone- and MAX2-mediated degradation, although SMAX1 D2 is relatively stable in the *smax1,2* background, suggesting that the full-length SMXL proteins or homologous oligomerization may influence D2 degradation^32^; loss of the RGKT motif in the D2 domain of D53 confers SL insensitivity in rice gain-of-function *d53* mutants.^28^^,^^29^^,^^32^^,^^47^ The D1M (D1+M) domain of SMAX1, SMXL7, or D53 directly interacts with AtD14/OsD14 or KAI2 but is not degraded like the D2 domain.^29^^,^^32^ Moreover, the direct interaction of OsD14 with d53, the SL-resistant mutant that retains wild-type ND1M, suggests an unresolved role of receptor-D1M binding in SL signaling.^28^^,^^29^ A cryo-electron microscopy (cryo-EM) structure of the SMAX1-ShMAX2-ShHTL7 complex confirms that the D2 domain binds both ShHTL7 and ShMAX2 to stabilize the complex.^24^ The N domain also contacts MAX2 in this complex, suggesting additional regions contribute to stabilization. Although the D1M domain is not visible in this cryo-EM structure, post-translational modification studies reveal phosphorylation sites in the M domain that protect SMXL6/7/8 from SL-triggered degradation.^48^

Taken together, SLs and KARs/KL regulate distinct physiological processes via targeted degradation of specific SMXL repressors, respectively, implying that the single E3 ubiquitin ligase MAX2 mediates ubiquitination of distinct SMXL clades. However, the molecular basis by which MAX2 recognizes diverse SMXL substrates in response to SL or KAR/KL signaling, and the identity of the hormone-responsive “input” domain in SMXL proteins, remain unresolved. For instance, the role of the D1M domain in receptor interaction remains unclear. Here, we investigate the molecular determinants of selective SMXL degradation induced by SLs and KARs/KL, focusing on how hormone signaling promotes assembly of substrate-specific signaling complexes.

## Results

### MAX2 specifically binds SMAX1 or SMXL7 via distinct receptors

**Figure 1:**
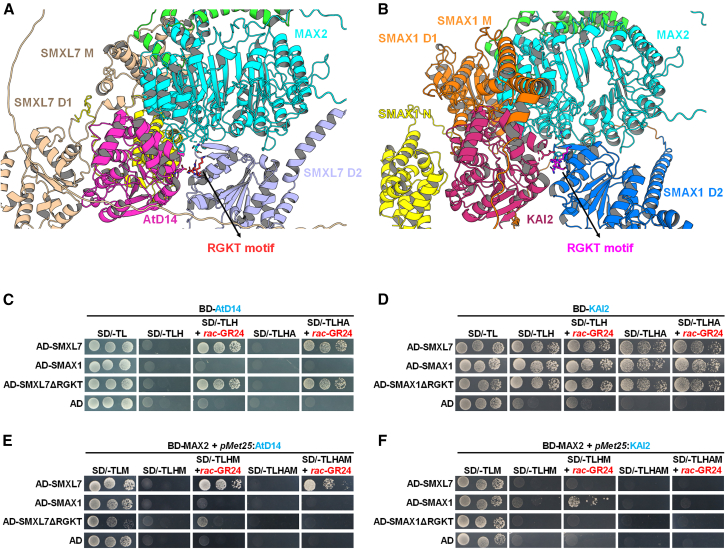
Receptor-dependent interaction between MAX2 and SMXL7 or SMAX1, with requirement of the SMXL RGKT motif (A and B) Structural models of ASK1-MAX2-AtD14-SMXL7 (A) and ASK1-MAX2-KAI2-SMAX1 (B) complexes predicted using AlphaFold server. RGKT motif is shown as sticks, labeled in red in the SMXL7 D2 domain (A) and labeled in magenta in the SMAX1 D2 domain (B). (C and D) Yeast two-hybrid (Y2H) assay measuring AtD14 or KAI2 interaction with SMXL proteins in the presence or absence of 5 μM *rac*-GR24. BD means GAL4 DNA-binding domain, AD means GAL4 activating domain. SD/-TLHA means selective medium SD/-Trp/-Leu/-His/-Ade. Three independent colonies represent serial dilution. (E and F) Yeast three-hybrid (Y3H) assay for detecting the formation of the MAX2-AtD14/KAI2-SMXLs complex in the presence or absence of 5 μM *rac*-GR24. AtD14 or KAI2 was cloned in the second cloning site under control of the *pMet25* promoter in pBridge and expressed in medium lacking methionine. SD/-TLHAM means selective medium SD/-Trp/-Leu/-His/-Ade/-Met. Three independent colonies represent serial dilution.

For SLs receptors, direct interaction of OsD14/AtD14 with D3/MAX2 is validated essential for D53 degradation and tillering regulation in rice and *Arabidopsis*, respectively.^23^^,^^49^ To further characterize the protein complexes mediating SMXL degradation, we predicted two ternary complex structures in *Arabidopsis* SLs/KARs signaling using the AlphaFold server: ASK1-MAX2-AtD14-SMXL7 and ASK1-MAX2-KAI2-SMAX1 (Figures 1A and 1B). Both predicted models show AtD14/KAI2 binds MAX2 at a position consistent with the previous reported AtD14-D3 crystal structure, OsD14-D3 cryo-EM structure in the presence of D53 and ShHTL7-ShMAX2 cryo-EM structure,^23^^,^^24^^,^^50^ while SMXL7/SMAX1 associates to form a ternary complex, consistent with the ShHTL7-ShMAX2-AtSMAX1 cryo-EM structure.^50^ The D2 domain plays a critical role: SMXL7 D2 interacts with both AtD14 and MAX2, and SMAX1 D2 binds KAI2 and MAX2. Notably, the RGKT motif in the D2 domain localizes to the ternary interface, highlighting the D2 domain and RGKT motif as key determinants of SL/KAR signaling complex formation.

To validate these models, we performed yeast two-hybrid (Y2H) assays. Consistent with previous work,^32^ AtD14 specifically binds SMXL7 only in the presence of *rac*-GR24 but not SMAX1 (Figure 1C). In contrast, KAI2 binds SMAX1 even without *rac*-GR24 and is also capable of interacting with SMXL7 (Figure 1D), consistent with the reported “sticky” interaction feature of KAI2.^32^^,^^34^^,^^51^ Importantly, RGKT-deletion (ΔRGKT) mutants of SMXL7 and SMAX1 do not affect receptor-repressor interactions (Figures 1C and 1D), similar to the OsD14-d53 interaction in rice,^28^^,^^29^ suggesting receptor binding alone is insufficient to trigger SMXL degradation.

Given MAX2’s essential role in SL/KAR signaling, we used yeast three-hybrid (Y3H) assays to evaluate MAX2-SMXL interactions in the presence of AtD14 or KAI2. Results show MAX2 specifically interacts with SMXL7 dependent on *rac*-GR24-activated AtD14 (Figure 1E), and with SMAX1 dependent on *rac*-GR24-activated KAI2 (Figure 1F). Unlike the hormone-independent KAI2-SMAX1 interaction, formation of the MAX2-KAI2-SMAX1 ternary complex strictly requires ligand, emphasizing the biological necessity of KARs/KL for SMAX1 degradation. Notably, ΔRGKT mutations in SMAX1 and SMXL7 nearly abolish MAX2 binding (Figures 1E and 1F), implying the RGKT motif (localized to the ternary interface in predicted structure models) mediates complex formation rather than acting as a ubiquitination site. Collectively, these data confirm that a ternary complex including MAX2, rather than receptor-SMXL interactions alone, is required for hormone-induced SMXL degradation.

### D1M and D2 domains of SMXL proteins mediate hormone responses

**Figure 2:**
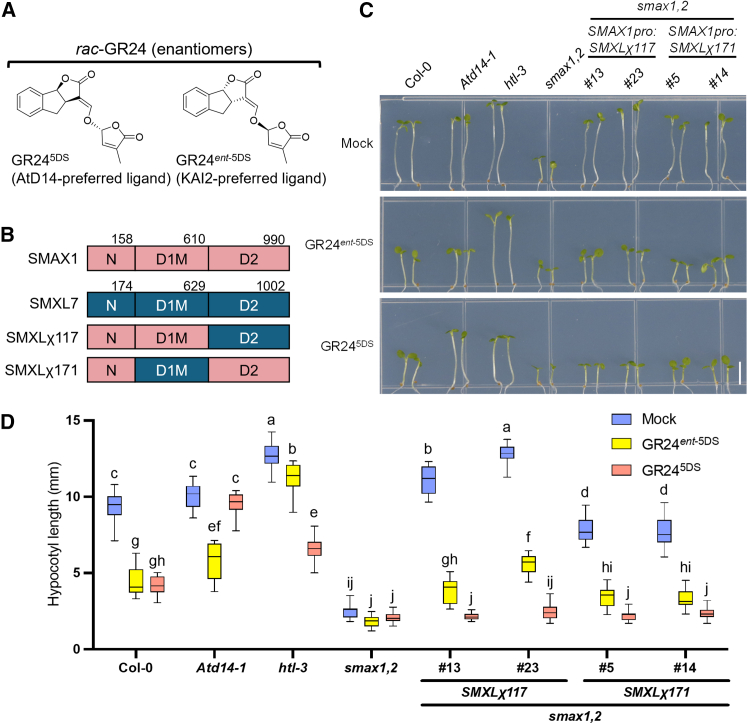
Chimeric SMXLs regulate hypocotyl elongation and responses to GR24 stereoisomers in *Arabidopsis* (A) Chemical structures of two stereoisomers that constitute *rac*-GR24. GR24^5DS^ is a AtD14-preferred ligand while GR24^*ent*−5DS^ is a KAI2-preferred ligand. (B) Schematic diagrams of SMXL domains and chimeric SMXL composition. (C) Representative phenotypes of 5-day-old *Arabidopsis* seedlings of Col-0, *Atd14-1*, *htl-3*, *smax1 smxl2* (*smax1,2)*, two individual transgene lines of *SMAX1* promoter driven *SMXLχ117* and *SMXLχ171* in smax1 smxl2 background. Seedlings were grown on 0.5× MS medium supplemented with 1 μM GR24^5DS^, GR24^*ent*−5DS^, or DMSO (Mock) under continuous red light. Scale bar, 5 mm. (D) Hypocotyl lengths of materials shown in (C) as mean ± SD (standard deviation). Statistics were analyzed using one-way ANOVA with Turkey test, *n* = 20, groups containing the same letters means their differences are not significant.

The N domain is regarded as the “output” domain controlling downstream physiological effects of SMXL proteins,^46^ but the “input” domains mediating hormone response and selectivity remain elusive. We performed chimeric complementation assays by swapping the D1M or D2 domains between SMAX1 and SMXL7 (Figure 2B). Chimeric SMXLs were expressed under the SMAX1 promoter in the *smax1 smxl2* double mutant background (to eliminate interference from endogenous SMAX1/SMXL2 in hypocotyl regulation).

Hypocotyl lengths of *SMAX1pro:SMXLχ171* and *SMAX1pro:SMXLχ117* complementation lines were restored similarly to wild-type levels, in contrast to the short hypocotyls of *smax1 smxl2* (Figures 2C and 2D), validating successful complementation and the functional role of the SMAX1 N domain. To distinguish hormone specificity, we tested GR24^5DS^ (SL analog) and GR24^*ent*−5DS^ (KAR analog), which together constitute the racemic mixture *rac*-GR24 (Figure 2A). Receptor specificity was confirmed: *Atd14-1* responded primarily to GR24^*ent*−5DS^ (ligand of KAI2), while *htl-3* (a commonly used *kai2* mutant, since AtHTL and KAI2 are alternative names of this gene) responded exclusively to GR24^5DS^ (ligand of AtD14) (Figures 2C and 2D). Hypocotyl length of both chimeric lines were significantly inhibited by both GR24^5DS^ and GR24^*ent*−5DS^, although GR24^5DS^ exhibits stronger inhibition (Figures 2C and 2D). Treatment with GR24^4DO^ (a potent and specific SMXL6 degradation inducer^35^) yielded similar hypocotyl elongation inhibition (Figures S1A and S1B).

These genetic data suggest the SMAX1 D2 domain in SMXLχ171 confers KAR responsiveness, while the SMXL7 D2 domain in SMXLχ117 mediates SL responses, consistent with the D2 domain’s role in degradation. Notably, SMXLχ117 lacks the SMAX1 D2 domain but retains KAR responsiveness. Since SMXL7 degradation is not induced by KARs,^30^^,^^31^ our results imply the SMAX1 D1M domain can facilitate KAR-triggered degradation when paired with the SMXL7 D2 domain.

### Two input domains facilitate hormone-induced SMXL degradation

Hormone responses of chimeric SMXL proteins reflect their degradation via SL or KAR/KL signaling. Confocal microscopy of complementation lines showed SMXLχ117-GFP and SMXLχ171-GFP localized to the root cap (consistent with *SMAX1* promoter activity; Figures S2A and S2B). Exogenous *rac*-GR24 induced degradation of both chimeric proteins, indicating they retain hormone-dependent turnover like native SMAX1 and SMXL7. Notably, SMXLχ171-GFP was almost degraded within 20 min while SMXLχ117-GFP required more time, aligning with distinct complementation phenotypes (Figures 2C and 2D).

**Figure 3:**
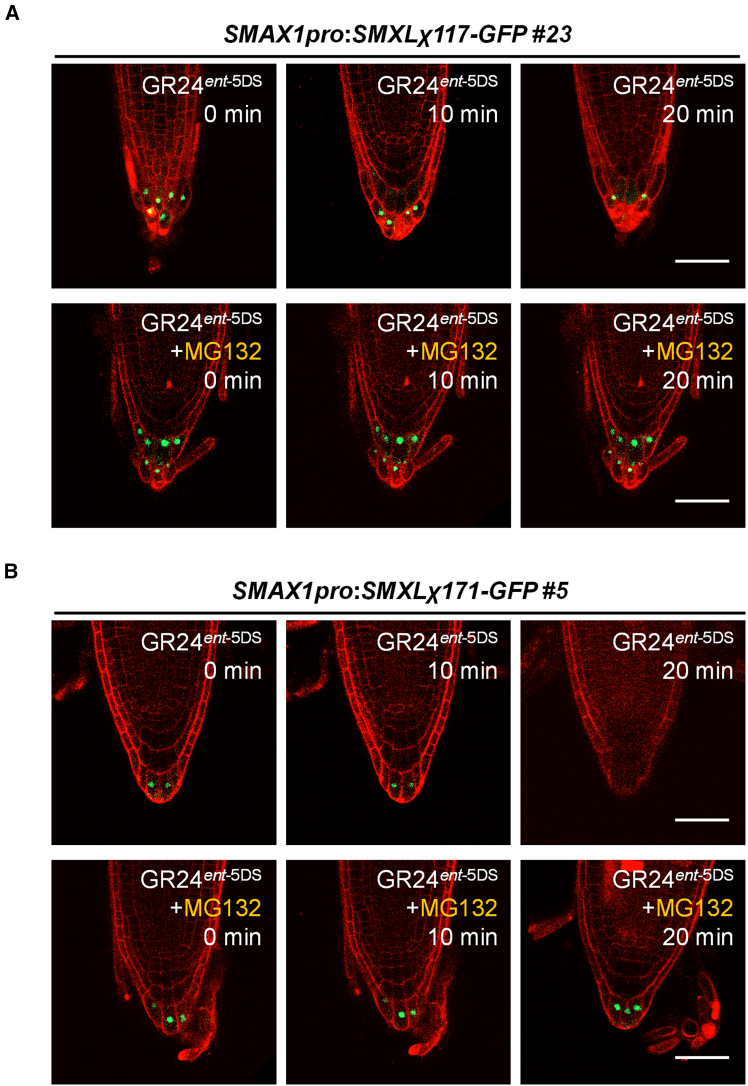
GR24^*ent*−5DS^ triggers chimeric SMXLs degradation *in vivo* (A) Roots of *SMAX1pro:SMXLχ117-GFP #23* complementation lines treated with 10 μM GR24^*ent*−5DS^ with or without 1-h pretreatment of 26S proteasome inhibitor MG132 at 50 μM. Images were taken at indicated times. Representative results were displayed from individual transgenic lines and more than three roots. Scale bar, 50 μm. (B) Roots of *SMAX1pro:SMXLχ171-GFP #5* complementation lines treated with 10 μM GR24^*ent*−5DS^ with or without 1-h pretreatment of 26S proteasome inhibitor MG132 at 50 μM. Images were taken at indicated times. Representative results were displayed from individual transgenic lines and more than three roots. Scale bar, 50 μm.

To define domains mediating degradation, we treated lines with GR24^*ent*−5DS^ (KAI2-specific agonist). GR24^*ent*−5DS^ promoted degradation of both chimeric SMXLs, which was inhibited by pre-treatment with the 26S proteasome inhibitor MG132 (Figures 3A and 3B), confirming proteasome-dependent turnover. Since SMXL7 is not degraded by KARs, these results suggest the SMAX1 D2 domain (in SMXLχ171) and SMAX1 D1M domain (in SMXLχ117) confer SMAX1-like degradation potential in the chimeric context. Moreover, there is residual SMXLχ117 after 20 min of GR24^*ent*−5DS^ treatments (Figure 3A), indicating a major role of the SMAX1 D2 domain in KARs-triggered degradation.

To further investigate receptor dependence, we performed cell-free degradation assays with purified SMXLχ117. Recombinant SMXLχ117 degraded rapidly within 20 min in total protein extracts from Col-0, *Atd14-1*, and *htl-3* supplemented with GR24^*ent*−5DS^, and degradation was suppressed by MG132 (Figure S4A). However, degradation was too rapid to assess individual receptor contributions. Incubation in *max2-3* extracts delayed but did not prevent SMXLχ117 degradation (Figure S4B), suggesting a MAX2-independent proteasomal degradation pathway. Shortening incubation times revealed that loss of AtD14 or KAI2 partially delayed degradation (Figure S5), indicating both receptors contribute to SMXLχ117 degradation. Nevertheless, the intrinsic instability of SMXLχ117 in cell-free systems complicated the delineation of receptor-dependent pathways.

### Receptor and E3 Ligase Specifically Bind the SMXL D2 Domain

**Figure 4:**
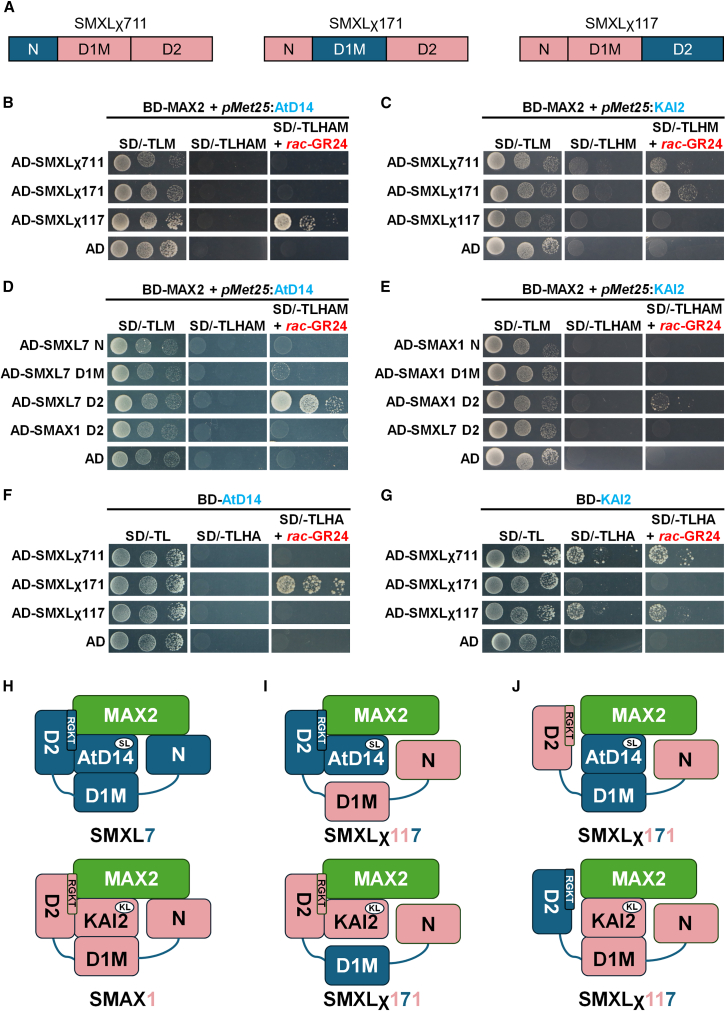
Strigolactone or Karrikin receptors assist MAX2 in specifically recognizing different SMXL proteins via the D2 domain (A) Schematic diagrams of chimeric SMXLs used in yeast hybrid assays. (B and C) Y3H assay for interactions between chimeric SMXL and MAX2 in the presence of AtD14 (B) or KAI2 (C) and 5 μM *rac*-GR24. BD means GAL4 DNA-binding domain, AD means GAL4 activating domain. AtD14 or KAI2 was cloned in the second cloning site under control of the *pMet25* promoter in pBridge and expressed in medium lacking methionine. SD/-TLHAM means selective medium SD/-Trp/-Leu/-His/-Ade/-Met. Three independent colonies represent serial dilution. (D and E) Y3H assay for interactions between each SMXL domain and MAX2 in the presence of AtD14 (D) or KAI2 (E) in the presence of 5 μM *rac*-GR24. Three independent colonies represent serial dilution. (F and G) Y2H assay to detect interactions between chimeric SMXLs and AtD14 (F) or KAI2 (G) in the presence of 5 μM *rac*-GR24. SD/-TLHA means selective medium SD/-Trp/-Leu/-His/-Ade. Three independent colonies represent serial dilution. (H–J) Models of the hormone signaling complex investigated in this study. AtD14 or KAI2 specifically targeting SMAX1 or SMXL7 through the ternary complex mediated by D2 domain and RGKT motif (H). Chimeric SMXL proteins possessing a compatible D2 domain could also form ternary complexes, which is validated by our Y3H results, although the D1M domain is not compatible (I). Chimeric SMXL proteins that do not contain a compatible D2 domain but actually respond to corresponding hormones. The interaction of the D1M domain with receptors might facilitate the targeted SMXL degradation.

To clarify the mechanism of chimeric SMXL ubiquitination and degradation, we analyzed protein-protein interactions via Y3H assays (Figure 4A). In the presence of hormone-activated AtD14, MAX2 interacted with SMXLχ117 (Figure 4B), indicating the SMXL7 D2 domain facilitates formation of the AtD14-MAX2-SMXLχ117 ternary complex. In contrast, the MAX2-KAI2 complex bound SMXLχ171 but not SMXLχ117 (Figure 4C), supporting the SMAX1 D2 domain’s role in this ternary complex. Collectively, these results confirm that the D2 domain is the primary mediator of ternary complex formation, consistent with physiological and degradation data.

We extended this analysis to individual SMXL domains. Y3H data showed AtD14 mediates MAX2 interaction with the SMXL7 D2 domain but not the SMAX1 D2 domain (Figure 4D), while KAI2 prefers the SMAX1 D2 domain over the SMXL7 D2 domain (Figure 4E), demonstrating the D2 domain confers selectivity for distinct hormone perception complexes. Additionally, ΔRGKT mutations in D2 domains abolished MAX2 interaction (Figure S6), validating the critical role of the D2 domain and RGKT motif in ternary complex formation.

In addition to the D2 domain, the D1M domain promotes chimeric SMXL ubiquitination/degradation and influences hypocotyl phenotypes, potentially via direct interaction with AtD14/KAI2 (Figures 2D and 3A). Y2H assays (without MAX2) showed AtD14 specifically binds SMXLχ171 but not SMXLχ117 in the presence of *rac*-GR24 (Figure 4F), consistent with SMXL7 D1M-AtD14 interaction. KAI2, although it still shows a hormone-independent binding in Y2H, actually binds to SMXLχ117 rather than SMXLχ171 (Figure 4G). Together, although the AtD14-mediated degradation of SMXLχ171 could be explained by that SMAX1 D2 domain is also degraded through SL signaling,^52^ KAI2-mediated degradation of SMXLχ117 cannot be attributed solely to the SMXL7 D2 domain. Rather, this likely involves KAI2-SMAX1 D1M binding, despite the fact that SMXLχ117 possesses a degradation-necessary D2 domain derived from SMXL7.

## Discussion

Our data provide insights into the SL/KAR signaling complexes consisting of a receptor (AtD14/KAI2), F box protein MAX2, and SMXL repressor (SMXL7/SMAX1). MAX2 interaction with SMXL substrates, mediated by distinct receptors, is essential for SMXL ubiquitination and degradation, as evidenced by abolished interaction between MAX2 and hormone-insensitive SMXL7ΔRGKT/SMAX1ΔRGKT mutants (Figures 1E and 1F). The higher complementation efficiency and protein stability of SMXLχ117 than SMXLχ171 implicated SMAX1 D2 as a key driver in KARs signaling (Figures 2C, 2D, 3A, and 3B). These findings also highlight the biological significance of hormones and receptors in guiding specific substrate selection by MAX2.

For the structural basis of ternary complex formation, the D2 domain of both SMAX1 and SMXL7 may interact with MAX2 (Figures 1A and 1B), but this interaction is weak in the absence of receptors (consistent with previous studies^49^^,^^53^ and our results; Figures 1E and 1F). When AtD14/KAI2/ShHTL7 is activated by hormone ligand, it undergoes conformational changes to form a stable complex with MAX2.^23^^,^^24^ The AtD14-SMXL7 D2 or KAI2-SMAX1 D2 interface might then enhance the MAX2-D2 interaction, stabilizing the ternary complex, supported by our Y3H data (Figures 4D and 4E), which is consistent with the cryo-EM structure showing the ShHTL7-SMAX1 D2 interface.^24^ Specifically, the RGKT motif is critical for this D2-MAX2-AtD14/KAI2 complex, as supported by Y3H assays (Figure S6) and AlphaFold models (Figures 1A and 1B). The RGKT motif likely constitutes the core of the MAX2-D2 interface for both SMAX1 and SMXL7, with receptors providing additional stabilization (Figure 4H). This aligns with predicted models showing K721/K702 (in the RGKT motif of SMXL7/SMAX1) forms polar contacts with MAX2 D610, and R719/R700 interacts with AtD14 D167 or KAI2 D165 (Figure S7). We further predicted the structures of ternary complexes targeting chimeric SMXL proteins, which supports the notion that D2 mediates the selective complex formation (Figure S8). Regarding D2-mediated substrate selectivity (Figures 4D and 4E), divergent AtD14-SMXL7 D2 and KAI2-SMAX1 D2 interfaces may exist but require further validation.

Chimeric analysis also revealed the D1M domain’s contribution to degradation (Figures 3 and 4). Y2H results suggest mechanisms for KAI2-mediated SMXLχ117 degradation and AtD14-mediated SMXLχ171 degradation (Figures 4F and 4G). However, SMXLχ117 is incompatible with the KAI2-mediated ternary complex model, and SMXLχ171 is incompatible with the AtD14-mediated model (Figure 4J). The D14-SMXL7 D1M or KAI2-SMAX1 D1M interface, validated in chimeric SMXLs (Figures 4F and 4G) and shown in predicted structural models (Figures 1A and 1B), may promote exposure of the D2 domain in potential SMXL7-SMXLχ117/SMXLχ171 heteromeric complexes, as SMXL7-SMXL5 heterodimer was reported improving SMXL7 stability against AtD14-triggered degradation.^45^ For example, hormone-induced KAI2-SMXLχ117 interaction may facilitate subsequent AtD14-mediated ternary complex formation (Figures 4I and 4J), even under low endogenous SL levels. Alternatively, GR24^*ent*−5DS^ may not be a strict KAI2 ligand and could activate AtD14 signaling to target SMXLχ117, as reported previously.^54^ Similar to the concerns surrounding ligand specificity, it remains possible that exogenous *rac*-GR24 may undergo unknown modification pathways within yeast cells, given that MAX2-KAI2-SMAX1 interaction is relatively weaker compared with the complex mediated by AtD14 (Figures 1E and 1F), Further genetic and biochemical evidence is needed to clarify whether degradation depends on AtD14, KAI2, or both.

In conclusion, our study demonstrates that D2 domain-guided ternary complexes are indispensable for the selective degradation of SMAX1 or SMXL7 in SL/KAR signaling, with RGKT being interaction residues required for ternary complex formation. Meanwhile, we also suggest that other domains (e.g., D1M) contribute to degradation in a D2 domain-dependent manner, providing further insights into SL/KAR signaling.

### Limitations of the study

This study reveals the molecular basis by which SLs or KARs target distinct SMXL repressors for degradation via receptor- and MAX2-mediated specific ternary complexes. The specific SMXL D2 domain and RGKT motif are essential for preferred ternary complex formation and degradation. However, the D1M domain, while not required for ternary complex formation, contributes to receptor binding and SMXL degradation, and its mechanism in regulating SMXL stability remains unclear. Additionally, the instability of SMXL proteins and SL hydrolysis-dependent ternary complexes hinders experimental structural determination of complexes involving full-length SMXL proteins.

## Resource availability

### Lead contact

Requests for further information and resources should be directed to and will be fulfilled by the lead contact, Ruifeng Yao (ryao@hnu.edu.cn).

### Materials availability

All materials generated in this study are available from the lead contact upon request. This study did not generate new unique reagents.

### Data and code availability

• Data reported in this paper will be shared by the lead contact upon request.
• This paper does not report original code.
• Any additional information required to reanalyze the data reported in this work is available from the lead contact upon request.

## Acknowledgments

We thank P. McCourt and S. Lumba (University of Toronto) for providing *htl-3*, S. Smith (University of Tasmania) for providing *Atd14-1*, and D. Nelson (University of California, Riverside) for providing *smax1-2 smxl2-1*. We thank P. Li and Y. Hao at the Analytical Instrumentation Center of Hunan University for assistance in confocal microscopy (LSM980). This work was supported by the 10.13039/501100012166National Key Research and Development Program of China (2022YFF1002000), Yuelushan Laboratory Breeding Program (YLS-2025-ZY01004, YLS-2025-ZY03001), the 10.13039/501100001809National Natural Science Foundation of China (32470340, 32270334), the 10.13039/501100002767Department of Science and Technology of Hunan Province, China (2023RC1050), and Hunan Science and Technology Innovation Plan (2025ZY1003).

## Author contributions

M.X. and R.Y. conceived and designed the study. M.X. and X.X. performed the experiments and data analyses. M.X. and R.Y. drafted the manuscript. M.Z., H.C., L.C., and R.Y. revised the manuscript.

## Declaration of interests

The authors declare no competing interests.

## STAR★Methods

### Key resources table

**Table:** 

REAGENT or RESOURCE	SOURCE	IDENTIFIER
**Antibodies**		

Mouse anti-His	Abmart	M20001
Goat Anti-Rabbit Mouse IgG-HRP	Abmart	M21003

**Bacterial and virus strains**		

DH5α Chemically Competent Cell	Tsingke	TSC-C14
Yeast strain AH109	Yu et al.^55^	N/A
BL21 (DE3) Chemically Competent Cell	Tsingke	TSC-E06
*Agrobacterium tumefaciens* strain GV3101	Tsingke	TSC-A01

**Biological samples**		

N/A	N/A	N/A

**Chemicals, peptides, and recombinant proteins**		

Phanta Max Super-Fidelity DNA Polymerase	Vazyme	P505
Murashige&Skoog Basal Medium with Vitamins	PhytoTech Labs	M519
MG-132	MCE	HY-13259
Ni-Charged Resin FF	GenScript	L00666-5
*rac*-GR24	Li et al.^54^	N/A
GR24^*ent*−5DS^	Li et al.^54^	N/A
GR24^5DS^	Li et al.^54^	N/A
GR24^4DO^	Li et al.^54^	N/A

**Experimental models: Organisms/strains**		

*Arabidopsis* Col-0	Lab stock	N/A
*smax1-2 smxl2-1 (smax1,2)*	Stanga et al.^27^	N/A
*Atd14-1*	Waters et al.^17^	N/A
*htl-3*	Toh et al.^56^	N/A
*SMAX1pro::SMXLχ117-GFP in smax1,2*	This study	N/A
*SMAX1pro::SMXLχ171-GFP in smax1,2*	This study	N/A

**Oligonucleotides**		

Primers are listed in Table S1	This study	N/A

**Recombinant DNA**		

*pCambia1300- SMAX1:SMXLχ117-GFP*	This study	N/A
*pCambia1300- SMAX1:SMXLχ171-GFP*	This study	N/A
*pBridge-AtD14*	Yu et al.^55^	N/A
*pBridge-KAI2*	This study	N/A
*pBridge-M1-ASK1-MAX2-M2-AtD14*	This study	N/A
*pBridge-M1-ASK1-MAX2-M2-KAI2*	This study	N/A
*pGADT7-SMXL7*	Yu et al.^55^	N/A
*pGADT7-SMXL7ΔRGKT*	This study	N/A
*pGADT7-SMAX1*	This study	N/A
*pGADT7-SMAX1ΔRGKT*	This study	N/A
*pGADT7- SMXLχ711*	This study	N/A
*pGADT7- SMXLχ171*	This study	N/A
*pGADT7- SMXLχ117*	This study	N/A
*pGADT7-SMXL7 N*	This study	N/A
*pGADT7-SMXL7 D1M*	This study	N/A
*pGADT7-SMXL7 D2*	This study	N/A
*pGADT7-SMXL7 D2ΔRGKT*	This study	N/A
*pGADT7-SMAX1 N*	This study	N/A
*pGADT7-SMAX1 D1M*	This study	N/A
*pGADT7-SMAX1 D2*	This study	N/A
*pGADT7-SMAX1 D2ΔRGKT*	This study	N/A
*pET28a-sumo-SMXLχ117*	This study	N/A

**Software and algorithms**		

GraphPad Prism 8	GraphPad	https://www.graphpad.com/
ImageJ	ImageJ	https://imagej.net/ij/download.html
PyMOL	N/A	https://pymol.org

### Experimental model and study participant details

#### Plant materials and growth conditions

*Arabidopsis thaliana* mutants *Atd14-1*, *htl-3*, and *smax1-2 smxl2-1* were described previously.^17^^,^^27^^,^^56^ Seeds were surface-sterilized, plated on 0.5× Murashige and Skoog (MS) solid medium containing 1% (w/v) sucrose, stratified at 4°C in darkness for 3 days, and germinated for 7 days under long-day conditions (16-h light/8-h dark) at 22°C. Seedlings were transferred to soil and grown under the same conditions.

### Method details

#### Plasmid construction and plant transformation

The *SMAX1* promoter (3 kb upstream of the *SMAX1* start codon) was amplified from Col-0 genomic DNA and cloned into a modified pCambia1300 binary vector with a C-terminal GFP tag. Coding sequences of N, D1M, and D2 domains of SMAX1 and SMXL7 (according to previous reported chimeric SMXLs^46^) were amplified with overlapping primers and assembled by overlap PCR, then cloned into the pCambia1300 vector between the *SMAX1* promoter and GFP.

*Agrobacterium tumefaciens* strain GV3101 carrying recombinant plasmids was transformed into *smax1,2* via floral dip. Transformed seeds were selected with hygromycin, and T3 homozygous lines with a single T-DNA insertion were used for phenotype analysis.

#### Hypocotyl elongation assay

Seeds were surface-sterilized, stratified at 4 °C in darkness for 3 days, and plated on 0.5× MS solid medium supplemented with chemicals or solvent control. Plates were treated with white light for 3 h, darkness for 21 h, and continuous red light (30 μmol m^-2^s^-1^) at 22°C for 4 days. Hypocotyl lengths were measured using ImageJ.

#### Yeast two- and three-hybrid assays

Full-length SMXLs, domains, and chimeras were cloned into pGADT7 (GAL4-AD fusion). *AtD14*, *KAI2*, and *ASK1-MAX2* (ASK1 fused to MAX2 for stabilization) were cloned into pBridge (GAL4-BD fusion). *AtD14/KAI2* was inserted into pBridge-ASK1-MAX2 within the second multiple cloning site (*Met25* promoter-driven). Yeast culture, transformation, and selection were performed as described previously.^55^

For yeast two-hybrid assay, plasmids were cotransformed into yeast strain AH109. Transformed cells were selected on SD/-Leu-Trp, and interactions were tested on SD/-Leu-Trp-His and SD/-Leu-Trp-His-Ade (with the absence of Ade applying stronger selection pressure) with *rac*-GR24 or solvent control.

For yeast three-hybrid assay, AH109 strains were streaked three times on SD/-Met, then with the same procedure in yeast two-hybrid assay. Interactions were tested on SD/-Leu-Trp-His-Met and SD/-Leu-Trp-His-Ade-Met with *rac*-GR24 or solvent control.

#### Protein degradation analysis in *Arabidopsis*

Sterilized seeds were plated on 0.5× MS solid medium and grown vertically for 4 days. Seedling roots were pretreated with 50 μM MG132 for 1 h or directly mounted on glass slides and soaked in 10 μM propidium iodide containing 10 μM GR24^*ent*-5DS^. Root cells were imaged with a Zeiss LSM980 confocal microscope (20 × lens). GFP was excited at 488 nm (emission: 510-540 nm); propidium iodide was excited at 543 nm (emission: 587-625 nm).

#### Recombinant protein expression and purification

His-sumo-SMXLχ117 was expressed in *E. coli* BL21(DE3) using pET28a vector. The bacterial cultures were grown in 2 L of LB broth at 37°C until the OD_600_ reached 0.6, cooled on ice for 10 minutes, and protein expression was induced by adding 0.2 mM IPTG for 16-20 hours at 16°C. The cultures were centrifuged at 4°C to pellet the cells, and all subsequent steps were performed at this temperature. The cells were lysed by sonication, and the lysate was centrifuged at 20,000 g for 1 hour to remove debris. The supernatant was then subjected to affinity purification using Ni-NTA agarose, with a binding buffer composed of 50 mM Tris-HCl (pH 8.0), 500 mM NaCl, and 4% (v/v) glycerol. Proteins were washed and eluted using binding buffer supplemented with 20-300 mM imidazole. The eluted proteins were concentrated by ultrafiltration for buffer exchange (binding buffer with 2 mM DTT) and concentrated to 10 mg/mL, then aliquoted, flash-frozen in liquid nitrogen and stored at -80°C.

#### Protein degradation analysis in cell-free systems

Cell-free degradation assays were performed as described previously.^9^ Total proteins from wild-type and mutant *Arabidopsis* were extracted (buffer: 50 mM Tris–HCl pH 7.5, 100 mM NaCl, 10% (v/v) glycerol, 20 mM β-mercaptoethanol, 0.1% (v/v) Tween 20). Purified His-sumo-SMXLχ117 (final concentration ∼50 μg/mL) was added to the abovementioned crude extracts with hormones/inhibitors and incubated at 22 °C for indicated times. Target protein levels were detected by immunoblotting with anti-His antibody; Ponceau S staining confirmed equal loading.

#### Prediction and visualization of protein complexes

SL/KAR signaling complex structures (ASK1-MAX2-AtD14-SMXL7 and ASK1-MAX2-KAI2-SMAX1) were predicted using the AlphaFold3 server (https://alphafoldserver.com). Previous studies have established that ASK1 is required for stabilizing MAX2/D3 during experimental structural determination.^23^ High-scoring models were analyzed with PyMOL to visualize complexes and RGKT motif-interacting residues.

### Quantification and statistical analysis

All data analysis was performed using GraphPad Prism 8 software, and ImageJ. Quantitative data are presented as mean ± SD (standard deviation), with detailed information provided in the figure legends. Statistical significance was determined using one-way ANOVA with Turkey’s test, and differences were considered significant at *P* < 0.05, as indicated by different letters in the graphs. All experiments involving measurements, imaging, and quantification were independently repeated at least three times, yielding consistent results.

## Supplemental information

Document S1. Figures S1–S8

Table S1. Primers used in this study
