# Supplementary material for: Divergent strigolactones or karrikins signaling is mediated by preferred AtD14/KAI2-MAX2-SMXL complex targeting specific SMXL domains
Source: iScience. 2026 Jun 1;29(6):116062. doi: 10.1016/j.isci.2026.116062 (PMC13233607; doi:10.1016/j.isci.2026.116062)
Supplement: Document S1. Figures S1–S8 [file mmc1.pdf]

iScience, Volume *29*

Supplemental information

Divergent strigolactones or karrikins signaling is mediated by preferred AtD14/KAI2-MAX2-SMXL complex targeting specific SMXL domains

Miao Xie, Xinbei Xu, Meng Zhang, Huihuang Chen, Li Chen, and Ruifeng Yao

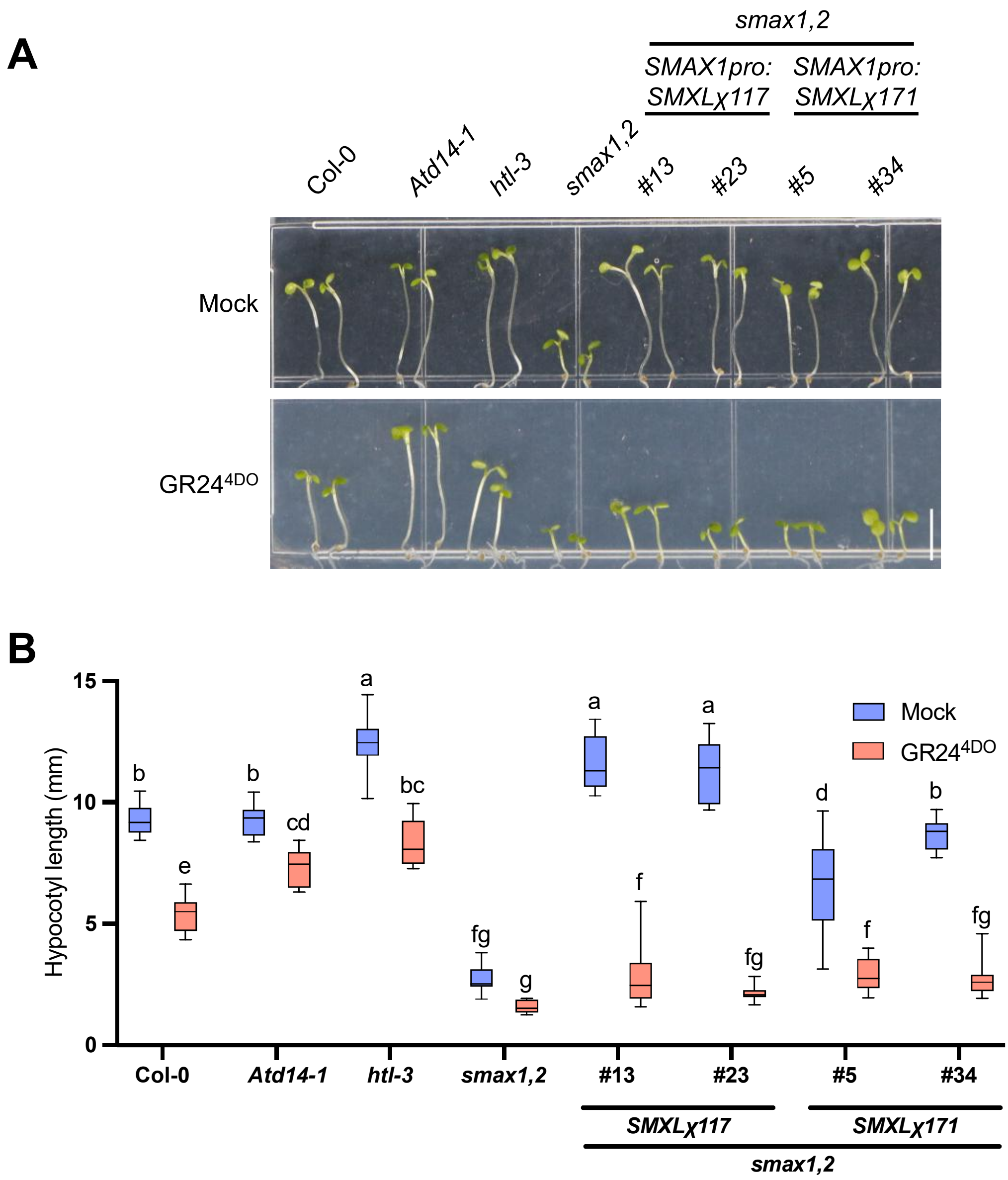

Figure S1. GR24^{4DO} inhibits hypocotyl elongation of chimeric SMXLs complementation lines, related to Figure 2

(A) Representative phenotypes of 5 days old Arabidopsis seedlings of Col-0, *Atd14-1*, *htl-3*, *smax1 smxl2* (*smax1,2)*, two individual transgene lines of *SMAX1* promoter driven *SMXLχ117* and *SMXLχ171* in *smax1 smxl2* background. Seedlings were grown on 0.5 × MS medium supplemented with 1 μM GR24^{4DO}, or DMSO (Mock) under continuous red light. Scale bar = 5 mm.

(B) Hypocotyl lengths of materials shown in (A) as mean ± SD (standard deviation). Statistics were analysed using one-way ANOVA with Turkey's test, n = 20, groups containing the same letters means their differences are not significant.

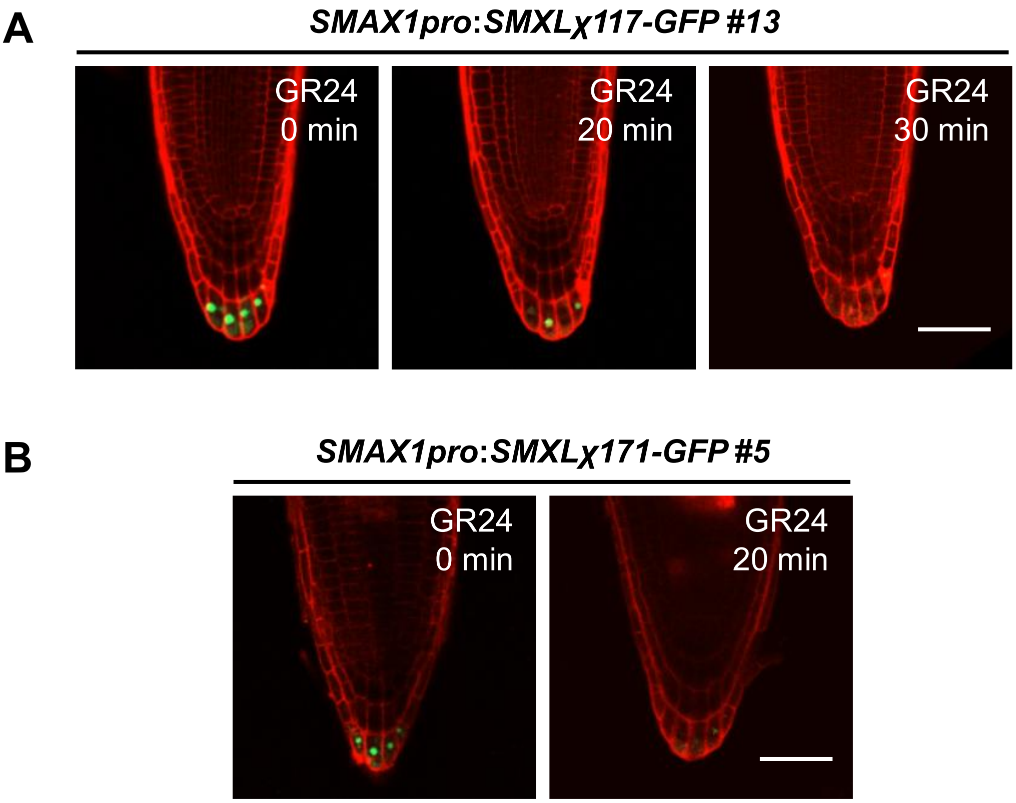

Figure S2. *rac*-GR24 triggers chimeric SMXLs degradation in vivo, related to Figure 3

(A) Roots of *SMAX1pro:SMXLχ117-GFP #13* complementation lines treated with 10 μM *rac*-GR24. Images were taken at indicated times. Representative results were displayed from individual transgenic lines and more than three roots, scale bar = 50 μm.

(B) Roots of *SMAX1pro:SMXLχ171-GFP #5* complementation lines treated with 10 μM *rac*-GR24. Images were taken at indicated times. Representative results were displayed from individual transgenic lines and more than three roots, scale bar = 50 μm.

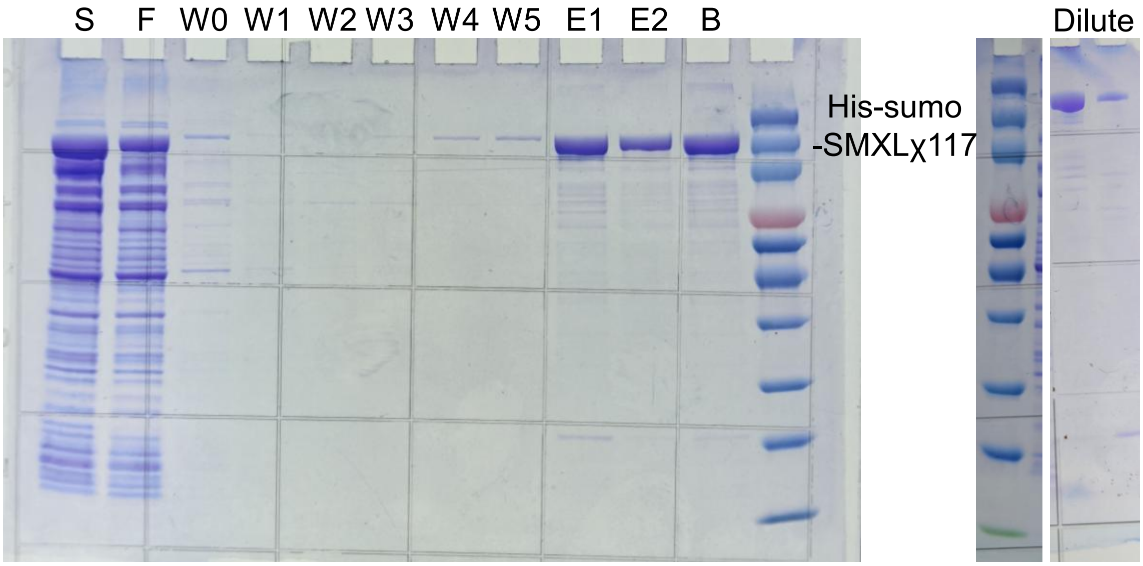

Figure S3. Purification of recombinant His-sumo-SMXLχ117, related to Figure 3
Coomassie blue staining results of *E. coli* expressed His-sumo-SMXLχ117 protein using Ni affinity beads. S means supernatant, F means flowthrough, W and E means gradient wash or elution with varying imidazole concentrations, B means proteins in Ni beads. Dilute suggests a 100-fold or 500-fold dilution of His-sumo-SMXLχ117 after buffer-exchange.

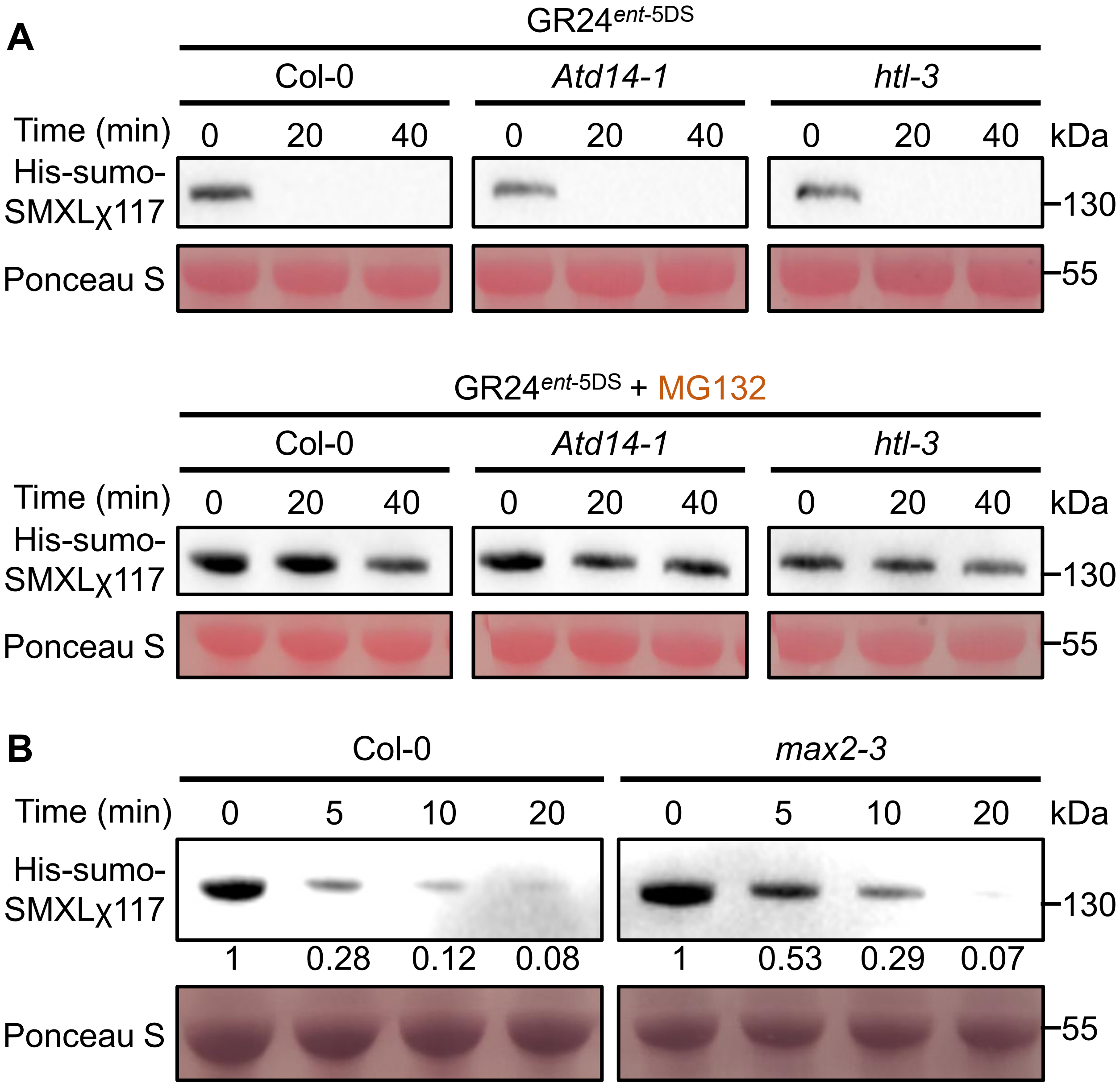

Figure S4. SMXLχ117 undergoes MAX2-dependent and -independent proteasomal degradation, related to Figure 3

(A) Purified His-sumo-SMXLχ117 protein was incubated in Col-0, *Atd14-1* or *htl-3* crude extracts supplemented with 5 μM GR24$^{ent\text{-}5DS}$ with or without 50 μM MG132. SMXLχ117 amounts at indicated time was detected using anti-His antibody. Membrane stained by ponceau S suggests equal amounts of crude extracts.

(B) Purified His-sumo-SMXLχ117 protein was incubated in Col-0 or *max2-3* crude extracts. Protein amounts was detected as in (A) and further quantified using ImageJ to show the delayed degradation in *max2-3.*

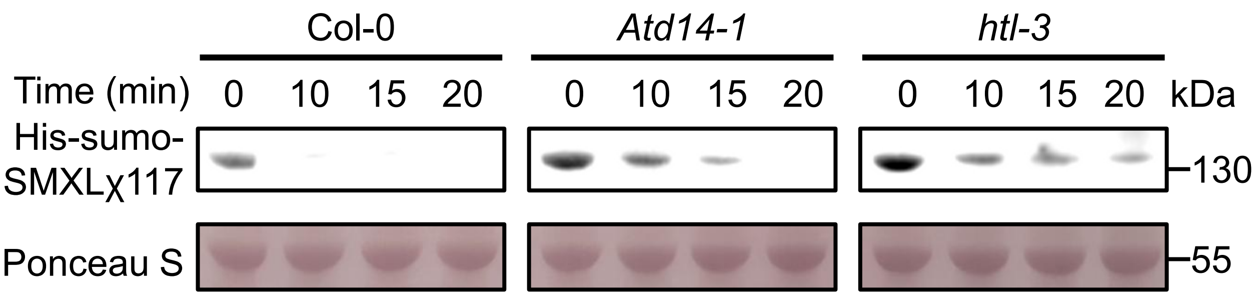

Figure S5. SMXLχ117 undergoes receptors-dependent and -independent degradation, related to Figure 3

Purified His-sumo-SMXLχ117 protein was incubated in Col-0, *Atd14-1* or *htl-3* crude extracts. SMXLχ117 amounts at indicated time was detected using anti-His antibody. Membrane stained by ponceau S suggests equal amounts of crude extracts.

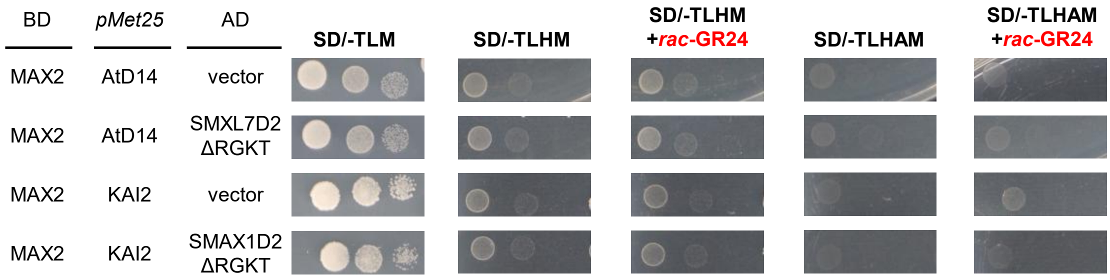

Figure S6. RGKT motif is essential for MAX2-D2 interaction, related to Figure 1 and 4
Y3H assay for interactions between SMXL D2 carrying RGKT-deletion mutant with MAX2 in the presence of AtD14 or KAI2 and 5 μM *rac*-GR24. BD means GAL4 DNA-binding domain, AD means GAL4 activating-domain. AtD14 or KAI2 was cloned in the second cloning site under control of *pMet25* promoter in *pBridge* and expressed in medium lacking methionine. SD/-TLHAM means selective medium SD/-Trp/-Leu/-His/-Ade/-Met. Three independent colonies represent serial dilution.

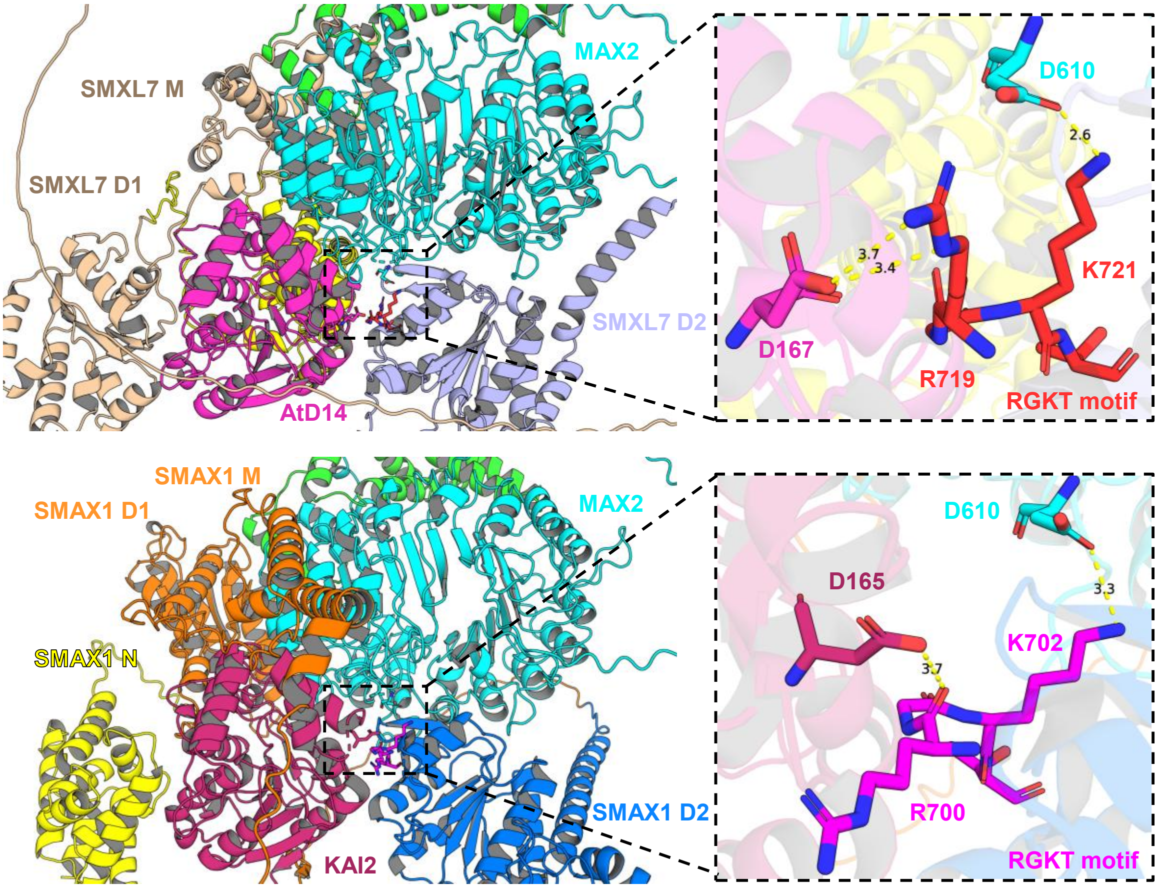

Figure S7. Details in predicted complex structure of ASK1-MAX2 with AtD14-SMXL7 or KAI2-SMAX1, related to Figure 1 and 4

Structural models of ASK1-MAX2-AtD14-SMXL7 and ASK1-MAX2-KAI2-SMAX1 as shown in Figure 1A and 1B. RGKT motif and their interacting residues: D167 of AtD14, D165 of KAI2 and D610 of MAX2, are shown in sticks. Potential polar contacts between O and N atoms from aspartic acid or lysine residues are labelled with yellow dash with distances.

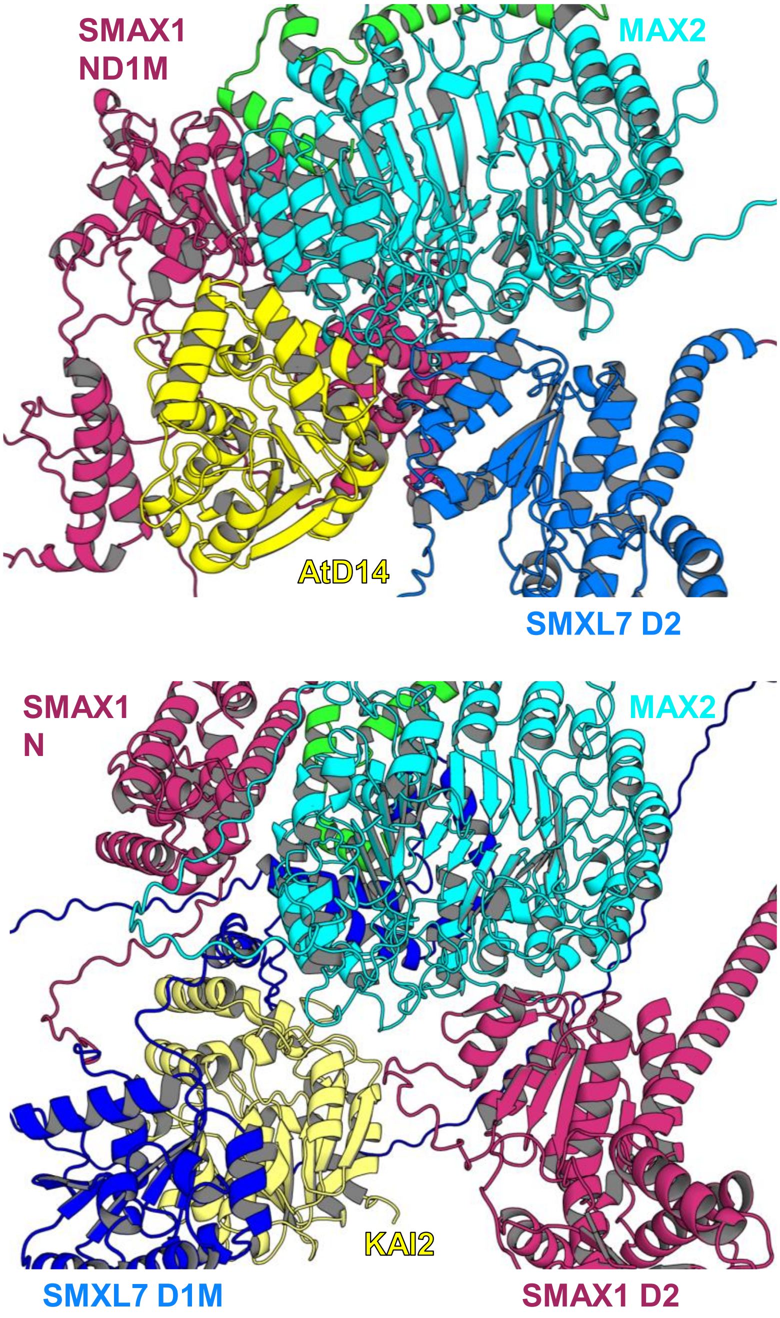

Figure S8. Predicted complex structure of ASK1-MAX2 with AtD14-SMXLχ117 or KAI2-SMXLχ171, related to Figure 1 and 4

Structural models of ASK1-MAX2-AtD14-SMXLχ117 and ASK1-MAX2-KAI2-SMXLχ171 using AlphaFold.
